# Supplementary material for: Conversion to belatacept after lung transplantation: Report of 10 cases
Source: PLoS One. 2023 Mar 15;18(3):e0281492. doi: 10.1371/journal.pone.0281492 (PMC10016650; doi:10.1371/journal.pone.0281492)
Supplement: S1 Data — (DOC) [file pone.0281492.s001.doc]

SUPPLEMENTAL DATA

**Immunosuppressive protocol:**

At Bichat hospital, no induction therapy was administered. Maintenance immunosuppressive (IS) therapy included tacrolimus (C0 trough level=8-12 ng/L), mycophenolate mofetil (2 g/day) and prednisone (5 mg/day). The surveillance protocol for acute cellular rejection (ACR) episodes and chronic lung allograft dysfunction (CLAD) was previously reported (1).

At Foch hospital, immunosuppression after LTx was based on an induction therapy with rabbit antithymocyte globulin (thymoglobulins; Genzyme, Lyon, France), 1.5 mg/kg per day for 3 days, except for: 1) cytomegalovirus (CMV)-infection–negative recipients with CMV infection–positive donors, 2) candidates colonized with *Burkholderia cepacia* or *Mycobacterium abcessus,*  3) recipient age > 65 years, 4) preoperative long-term administration of corticosteroids > 10-15 mg/day, and 5) high probability of postoperative extracorporeal membrane oxygenation, receiving basiliximab (Simulect; Novartis, Basel, Switzerland) for induction therapy. All lung-transplant recipients received maintenance immunosuppressive (IS) therapy with tacrolimus, mycophenolate mofetil (2 g/day) and prednisolone (500 mg intravenous [i.v.] methylprednisolone before surgery and before reperfusion of the graft; 1mg/kg/day on the following days and thereafter replaced by oral prednisone, which was progressively tapered to 0.1 mg/kg/day after 12 weeks). Tacrolimus monitoring involved the pre-dose concentration (C0), with target pre-dose concentration levels of 8 to 12 ng/mL.

In both centers, ACR episodes > A1 grade were treated with IV methylprednisolone (15 mg/kg/day) for 3 days, then an oral taper of prednisolone. Steroid-resistant ACR episodes were treated with rabbit antithymocyte globulin therapy (2.5 mg/kg/day for 5 days) in case of failure of a 3-day course of IV methylprednisolone (15 mg/kg/day). Proven and probable antibody-mediated rejection (AMR) (2) was treated with plasmapheresis (5 sessions), rituximab (375 mg/m2), and IV immunoglobulin (2 g/kg).

**Prophylaxis of opportunistic infections:**

Prophylactic treatment against pneumocystosis was systematically used in all patients (in both centers), with Trimetoprim/Sulfamethoxasone (800mg): 800 mg 3 times a week, or atovaquone 750 mg x 2/day in case of side effects of Trimetoprim/Sulfamethoxasone, lifelong from day 0 of LTx.

Prophylactic treatment of CMV infection at Bichat hospital included Rovalcyte: 900 mg/day from day 0 post LTx to 6 months in absence of CMV mismatch (for D+/R+ or D-/R+ status), or to 12 months post-LTx in case of CMV mismatch (D+/R-). At Foch hospital, same prophylactic treatment was used, except in patients with CMV D+/R+ or D-/R+ status, for who Zelitrex 2000 mg x 4/day was used during the first 6 months. In both centers, in case of negative CMV status (D-/R-), a prophylactic treatment with Zelitrex 1000 mg x3/day was given within the first 3 months.”

**HLA typing and cross-matching**

In both centers, human leukocyte antigen (HLA) typing of recipients involved molecular biology (Innolipa HLA typing kit; Innogenetics, Gent, Belgium). For all LTx donors, HLA-A/B/DR/DQ tissue typing involved the microlymphocytotoxicity technique with tissue-typing trays (One Lambda, Canoga Park, CA), and was controlled by molecular biology (3). Within 24 hours of transplantation, LTx recipients underwent auto- and allo-anti-human globulin complement-dependent cytotoxicity (AHG-CDC) T-cell and CDC B-cell crossmatching with and without dithiotreitol reduction, using current (day of transplantation) and historic sera (3). Patients were screened for anti-HLA Abs by single-antigen flow-beads (SAFB) Luminex assay (LSA, One Lambda, Canoga Park, CA) at day 0, and months 1, 6, 12, and also in case of clinical event in the post-LTx period.

**Monitoring protocols of patients**.

During follow-up of patients, surveillance of ACR and AMR episodes was performed with systematic transbronchial biopsies (TBBx) (months 1, 2, 3, 6, 9, 12 post-LTx) at Foch center, and/or in case of clinical, physiological or radiographic changes at Bichat center. During fibroptic endoscopy, bronchoalveolar lavage fluid (BALF) was systematically tested to determine the presence of bacteria, viruses and fungi (4). Multiplex polymerase chain reaction (PCR) assay was used to detect respiratory viruses in BALF. All TBBx specimens were systematically assessed for ACR, and an ACR episode was diagnosed histologically and graded according to the International Society for Heart and Lung Transplantation (ISHLT) criteria(5). An AR score was defined by the number of biopsy-proven cellular AR episodes, graded according to the International Society for Heart and Lung Transplantation (ISHLT) criteria(5), during the first 12 months after LTx (6). Diagnosis of AMR was retrospectively made according to criteria of ISHLT(2). CLAD was classified into two clinical phenotypes: bronchiolitis obliterans syndrome (BOS)(7, 8), defined by the classical ISHLT definition as restrictive allograft syndrome (RAS)(9) or mixed phenotype.

Primary graft dysfunction (PGD) was diagnosed and graded according to ISHLT criteria (10).

**Patients data**

Follow-up after CNI-belatacept conversion included lung function test (forced expiratory volume in 1 second [FEV1], forced vital capacity [CV]), renal function (estimated glomerular filtration rate and serum creatinine level), histologic assessment of monthly transbronchial biopsies (TBBx ) during the next 3 months after starting belatacept (only performed at Bichat center), and infectious episodes until last available follow-up of all patients.

Glomerular filtration rate (GFR) was estimated by using the CKD-EPI (Chronic Kidney Disease-Epidemiology Collaboration) creatinine equation (13).

This study was approved by an institution ethics committee (IRB00012437), and conducted in accordance with good clinical practices and the recommendations concerning human research contained in the Declaration of Helsinki. All patients gave their informed consent to be included in the study.

**Belatacept protocol**

The dose regimen of belatacept was the “less-intensive” (LI) belatacept regimen reported in kidney-Tx (11, 12), as follows in all patients: 10 mg/kg on days 1 and 5; then at the end of weeks 2, 4, 8, and 12; next, then 5 mg/kg starting on week 16; then every 4 weeks thereafter. In the n=7 patients of the Bichat center with a belatacept-based IS regimen without CNI, the CNI (Tacrolimus or ciclosporine) was continued for 2 weeks, with reduction of initial target blood level of 50% the first week, of 30% the second week, then stopped on day 14. Mycophenolate mofetil and corticosteroids were continued according to the current IS protocol. In the n=3 patients of the Foch center with a CNI-sparing belatacept IS regimen, the dosage of CNI (Tacrolimus or ciclosporine) was reduced and adjusted to new target blood levels (2-3 ng/ml for Tacrolimus T0, and 40-70 ng/ml for ciclosporine T0).

**Renal outcome after belatacept**

After starting belatacept, mean creatinine value of the 10 patients under belatacept increased from 265±112 (n=10) to 156±62 (n=10) at 1 month (p=0.005), to 135±54 (n=9) at 3 months (p=0.003), 127±61 (n=8) at 6 months (p=0.005), and 126±65 (n=9) at last-follow-up under belatacept (p=0.001).

REFERENCES

1. Brugiere O, Suberbielle C, Thabut G, Lhuillier E, Dauriat G, Metivier AC, Gautreau C, Charron D, Mal H, Parquin F, Stern M. Lung transplantation in patients with pretransplantation donor-specific antibodies detected by Luminex assay. *Transplantation* 2013; 95: 761-765.

2. Levine DJ, Glanville AR, Aboyoun C, Belperio J, Benden C, Berry GJ, Hachem R, Hayes D, Jr., Neil D, Reinsmoen NL, Snyder LD, Sweet S, Tyan D, Verleden G, Westall G, Yusen RD, Zamora M, Zeevi A. Antibody-mediated rejection of the lung: A consensus report of the International Society for Heart and Lung Transplantation. *J Heart Lung Transplant* 2016; 35: 397-406.

3. Brugiere O, Roux A, Le Pavec J, Sroussi D, Parquin F, Pradere P, Dupin C, Bunel V, Mourin G, Jebrak G, Dauriat G, Castier Y, Mordant P, Lortat-Jacob B, Jean-Baptiste S, Mal H, Suberbielle C, Gautreau C, Caillat-Zucman S, Cazes A, Thabut G, Taupin JL. Role of C1q-binding anti-HLA antibodies as a predictor of lung allograft outcome. *Eur Respir J* 2018; 52.

4. Brugiere O, Thabut G, Mal H, Marceau A, Dauriat G, Marrash-Chahla R, Castier Y, Leseche G, Colombat M, Fournier M. Exhaled NO may predict the decline in lung function in bronchiolitis obliterans syndrome. *Eur Respir J* 2005; 25: 813-819.

5. Stewart S, Fishbein MC, Snell GI, Berry GJ, Boehler A, Burke MM, Glanville A, Gould FK, Magro C, Marboe CC, McNeil KD, Reed EF, Reinsmoen NL, Scott JP, Studer SM, Tazelaar HD, Wallwork JL, Westall G, Zamora MR, Zeevi A, Yousem SA. Revision of the 1996 working formulation for the standardization of nomenclature in the diagnosis of lung rejection. *J Heart Lung Transplant* 2007; 26: 1229-1242.

6. Swarup R, Allenspach LL, Nemeh HW, Stagner LD, Betensley AD. Timing of basiliximab induction and development of acute rejection in lung transplant patients. *J Heart Lung Transplant* 2011; 30: 1228-1235.

7. Meyer KC, Raghu G, Verleden GM, Corris PA, Aurora P, Wilson KC, Brozek J, Glanville AR, Committee IAEBTF, Committee IAEBTF. An international ISHLT/ATS/ERS clinical practice guideline: diagnosis and management of bronchiolitis obliterans syndrome. *Eur Respir J* 2014; 44: 1479-1503.

8. Verleden GM, Glanville AR, Lease ED, Fisher AJ, Calabrese F, Corris PA, Ensor CR, Gottlieb J, Hachem RR, Lama V, Martinu T, Neil DAH, Singer LG, Snell G, Vos R. Chronic lung allograft dysfunction: Definition, diagnostic criteria, and approaches to treatment-A consensus report from the Pulmonary Council of the ISHLT. *J Heart Lung Transplant* 2019; 38: 493-503.

9. Glanville AR, Verleden GM, Todd JL, Benden C, Calabrese F, Gottlieb J, Hachem RR, Levine D, Meloni F, Palmer SM, Roman A, Sato M, Singer LG, Tokman S, Verleden SE, von der Thusen J, Vos R, Snell G. Chronic lung allograft dysfunction: Definition and update of restrictive allograft syndrome-A consensus report from the Pulmonary Council of the ISHLT. *J Heart Lung Transplant* 2019; 38: 483-492.

10. Christie JD, Carby M, Bag R, Corris P, Hertz M, Weill D, Dysfunction IWGoPLG. Report of the ISHLT Working Group on Primary Lung Graft Dysfunction part II: definition. A consensus statement of the International Society for Heart and Lung Transplantation. *J Heart Lung Transplant* 2005; 24: 1454-1459.

11. Vincenti F, Charpentier B, Vanrenterghem Y, Rostaing L, Bresnahan B, Darji P, Massari P, Mondragon-Ramirez GA, Agarwal M, Di Russo G, Lin CS, Garg P, Larsen CP. A phase III study of belatacept-based immunosuppression regimens versus cyclosporine in renal transplant recipients (BENEFIT study). *Am J Transplant* 2010; 10: 535-546.

12. Perez CP, Patel N, Mardis CR, Meadows HB, Taber DJ, Pilch NA. Belatacept in Solid Organ Transplant: Review of Current Literature Across Transplant Types. *Transplantation* 2018; 102: 1440-1452.
